# Repositioning salicylanilide anthelmintic drugs to treat adenovirus infections

**DOI:** 10.1038/s41598-018-37290-3

**Published:** 2019-01-09

**Authors:** José A. Marrugal-Lorenzo, Ana Serna-Gallego, Judith Berastegui-Cabrera, Jerónimo Pachón, Javier Sánchez-Céspedes

**Affiliations:** 1 Clinical Unit of Infectious Diseases, Microbiology, and Preventive Medicine, Institute of Biomedicine of Seville (IBiS), University Hospital Virgen del Rocío/CSIC/University of Seville, 41013 Seville, Spain; 2 Department of Medicine, University of Seville, 41009 Seville, Spain

## Abstract

The repositioning of drugs already approved by regulatory agencies for other indications is an emerging alternative for the development of new antimicrobial therapies. The repositioning process involves lower risks and costs than the de novo development of novel antimicrobial drugs. Currently, infections by adenovirus show a steady increment with a high clinical impact in immunosuppressed and immunocompetent patients. The lack of a safe and efficacious drug to treat these infections supports the search for new antiviral drugs. Here we evaluated the anti-adenovirus activity of niclosanide, oxyclozanide, and rafoxanide, three salicylanilide anthelmintic drugs. Also, we carried out the cytotoxicity evaluation and partial characterization of the mechanism of action of these drugs. The salicylanilide anthelmintic drugs showed significant anti-adenovirus activity at low micromolar concentrations with little cytotoxicity. Moreover, our mechanistic assays suggest differences in the way the drugs exert anti-adenovirus activity. Niclosamide and rafoxanide target transport of the HAdV particle from the endosome to the nuclear envelope, whilst oxyclozanide specifically targets adenovirus immediately early gene E1A transcription. Data suggests that the studied salicylanilide anthelmintic drugs could be suitable for further clinical evaluation for the development of new antiviral drugs to treat infections by adenovirus in immunosuppressed patients and in immunocompetent individuals with community-acquired pneumonia.

## Introduction

Human adenovirus (HAdV) can be classified into more than 60 serotypes divided into seven species (HAdV-A to -G)^1^. The important clinical impact of HAdV infections in immunosuppressed patients is well documented^2–5^. Additionally, although the incidence of HAdV in immunocompetent individuals with community-acquired pneumonia (CAP) appears to be low, the current availability of molecular techniques of diagnosis has allowed the identification of HAdV as an important etiologic agent of both occasional cases and outbreaks of CAP in healthy individuals^6–9^. Unfortunately, currently there are not approved antiviral drugs to specifically treat HAdV infections and the clinically available broad-spectrum antivirals show no satisfactory therapeutic response in terms of efficacy or safety against HAdV infections^10^.

The repositioning of drugs already approved by regulatory agencies for other indications is emerging as an alternative for the development of new antimicrobial therapies, a process that involve lower risks and costs than the de novo development of novel antimicrobial drugs^11–13^. In this way, niclosamide (NIC), a US Food and Drug Administration (FDA)-approved drug for treating helminthic infections, has previously shown antiviral activity against a broad range of pH-dependent viruses^14–17^. NIC was postulated to block virus entry at low micromolar concentrations targeting endosomal acidification. However, a recent publication by Xu *et al*. suggested that NIC was probably targeting viral replication^14,17^.

**Figure 1:**
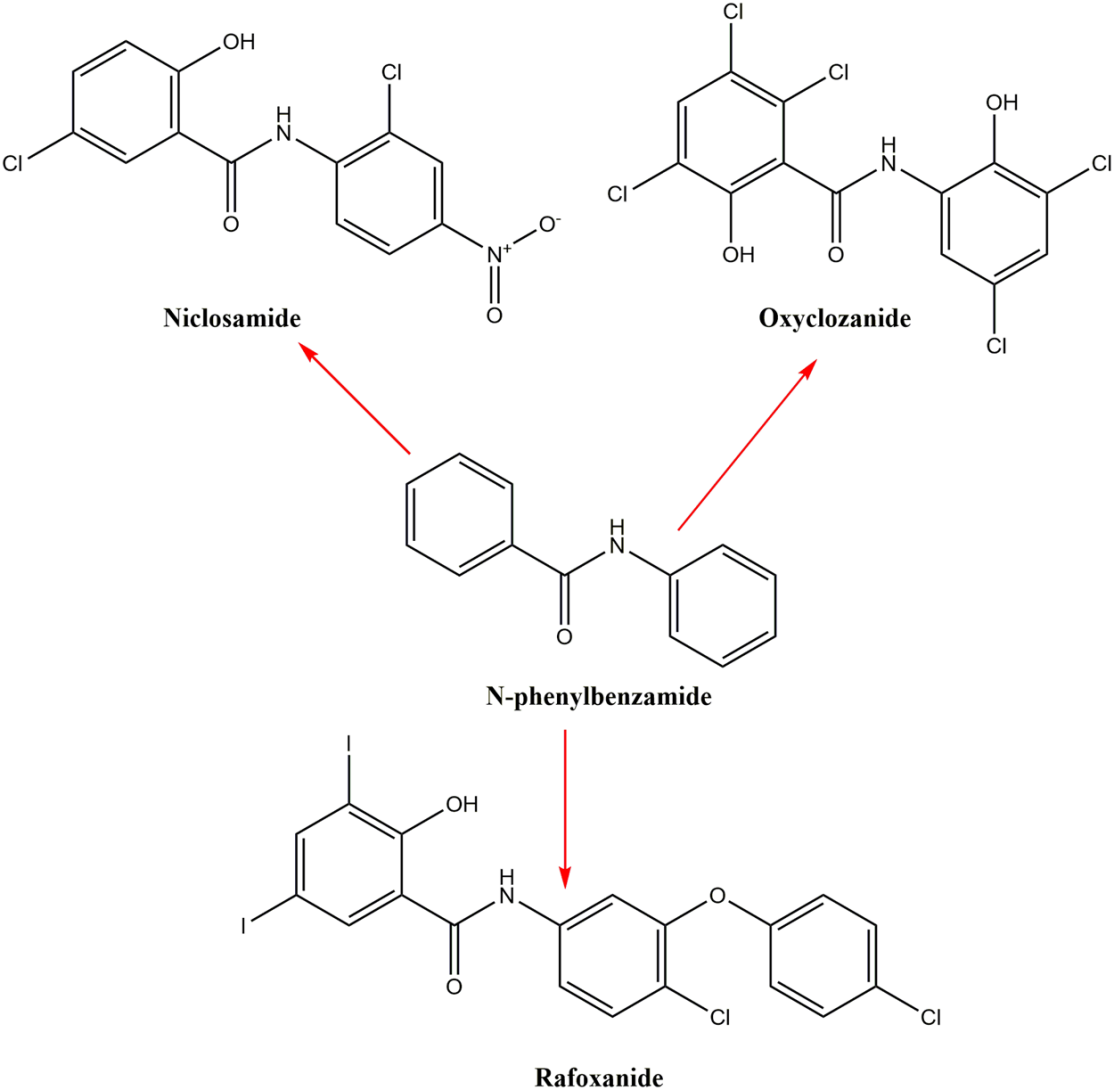
Molecular structure of salicylanilide anthelmintic drugs.

In this study, we evaluated the anti-HAdV activity of NIC and the other two commercially available salicylanilide anthelmintic drugs, oxyclozanide (OXY), and rafoxanide (RAF). All of the drugs examined share a central structure with a core of N-phenyl benzamide (Fig. 1); we examined their activity to evaluate their potential as repositioned drugs to treat HAdV infections.

## Results

### Anti-adenovirus activity of salicylanilide anthelmintic drugs

**Figure 2:**
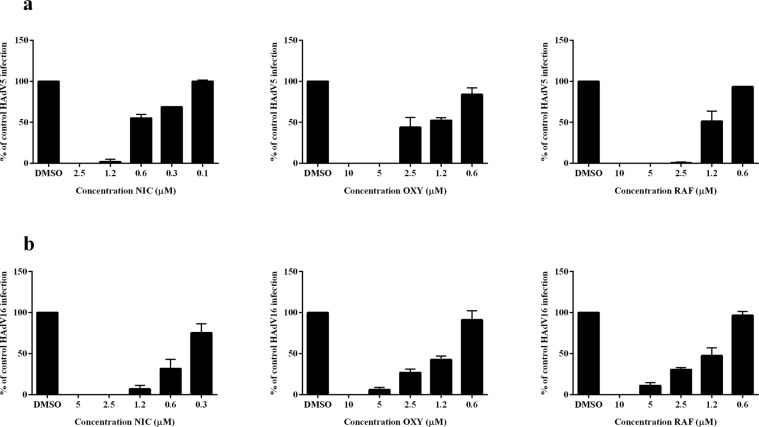
Inhibitory activity of NIC, OXY and RAF at low MOI. Dose-dependent activity of NIC, OXY and RAF against HAdV5 (**a**) and HAd16 (**b**) in a plaque assay at low MOI (0.06 vp/cell) using the 293β5 cell line. For all panels, the DMSO control is a negative control consisting of cells infected at the same MOI in the absence of drugs. Results represent means ± SD of triplicate samples from three independent experiments.

In initial studies, we determined the inhibitory concentrations of the three anthelmintic drugs against HAdV in a plaque assay measured as the percentage of plaque formation inhibition compared to a control cells infected in the absence of drugs. The three salicylanilide anthelmintic drugs showed a dose-dependent anti-HAdV activity against both HAdV5 and HAdV16, with 100% inhibition of plaques formation at 1.25, 5 and 2.5 μM for NIC, OXY and RAF, respectively (Fig. 2a,b). The IC_50_ values for the three anthelmintic drugs are summarized in Table 1. We also evaluated whether these salicylanilide anthelmintic drugs were able to inhibit virus production using HAdV5, HAdV16 and HAdV19 yield reduction assays. Treatment with NIC, OXY and RAF was associated with overall reductions in virus yield from 10 to 186-fold (Table 1).

**Table 1:** IC_50_, CC_50_, SI values and virus yield reduction for NIC, OXY and RAF.

	CC_50_ (µM)	IC_50_ (µM)		Selectivity Index (SI)		Virus Yield Fold-reduction (TCID_50_/ml compound *vs*. DMSO)		
		HAdV5-GFP	HAdV16-GFP	HAdV5	HAdV16	HAdV5-WT	HAdV16-WT	HAdV19-WT
NIC	3.30 ± 0.4	0.6 ± 0.05	0.45 ± 0.1	5.5	7.3	82 ± 35 (5.60e + 8/3.90e + 10)	21 ± 0 (4.64e + 7/1.00e + 09)	46 ± 25 (8.61e + 1/2.76e + 03)
OXY	78.93 ± 2.8	2.3 ± 0.7	1.26 ± 0.0	34.3	62.6	10 ± 2 (3.90e + 9/3.90e + 10)	26 ± 7 (3.90e + 7/1.00e + 09)	59 ± 0 (4.64e + 1/2.76e + 03)
RAF	48.89 ± 9.0	1.3 ± 0.1	1.38 ± 0.2	37.6	35.4	186 ± 58 (5.98e + 8/1.10E + 11)	42 ± 6 (2.40e + 7/1.00e + 09)	40 ± 16 (4.15e + 1/2.76e + 03)

Assays to determine CC_50_ and IC_50_ (MOI 0.06 vp/cell) values were carried out using the 293β5 cell line. For the HAdV yield reduction assay the cell line used was A549 and the MOI of HAdV was 100 vp/cell.

**Figure 3:**
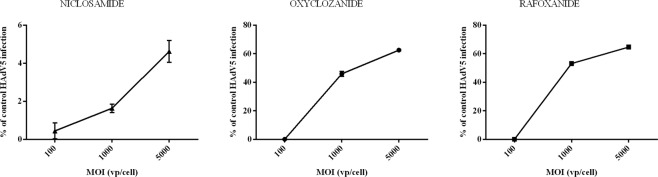
MOI dependence assay of NIC, OXY and RAF. % of HAdV wt de novo synthesis of hexon DNA in presence of NIC (5 μM), OXY (25 μM) and RAF (25 μM) relative to the control with DMSO, measured by quantitative PCR. The results represent means ± SD of triplicate samples from three independent experiments.

In an assay measuring antiviral activity of the three anthelmintic drugs as a function of MOI, inhibition was inversely proportional to the number of input particles (Fig. 3). While they were also inhibitory at high MOI, the relationship between the number of infecting HAdV particles and the drugs concentration is less marked, as expected.

The cellular cytotoxicity of the salicylanilide drugs was also analyzed. The CC_50_ values for these molecules were in all cases significantly higher than the IC_50_ concentrations required for inhibition in our antiviral activity and mechanistic assays for both 293β5 cells (Table 1) and A549 cells (22.9 ± 9.8 µM, 76.1 ± 14.4 µM and 80.6 ± 34.7 µM for NIC, OXY and RAF, respectively).

### **OXY inhibits a Late** Step in HAdV replicative cycle

**Figure 4:**
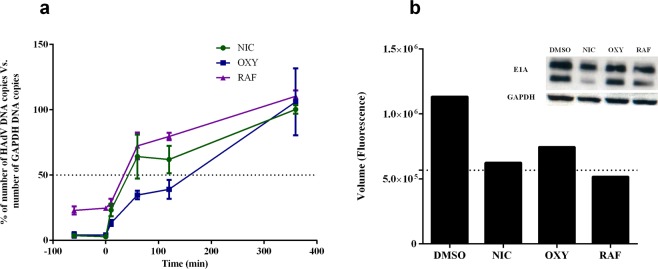
Impact on HAdV entry. HAdV5 infection of A549 cells was measured as a function of the time of addition of the three anthelmintic drugs. Negative time points refer to incubation of virus with the drugs in the absence of cells at 4 °C and time 0 is the start of incubation at 37 °C. Results are expressed as the relative copy number of HAdV DNA and the copy number of the GAPDH gene, in both cases normalized to a control of infection in the absence of anthelmintic drug (but in presence of DMSO) and are presented as the mean ± SD from triplicate assays (**a**).Western-blot for HAdV protein E1A at 6 hpi in the presence of the three anthelmintic drugs compared to a control in presence of DMSO (**b**). Full-length scans of Western blotting films are shown in Supplementary Fig. S1.

As the first step toward identifying the specific step in the HAdV replicative cycle that was inhibited by these drugs, we measured the time dependence of the three anthelmintic drugs addition on their ability to block HAdV infection. A previous report showed biochemically that HAdV viral particles were internalized within 5 min of binding and reach the nuclear pore after 45 min^18^. Our results demonstrated that NIC, OXY and RAF exhibited a time-dependent decrease in their inhibitory activity (Fig. 4a). All of them showed high inhibition of HAdV infection when added at the beginning of the 60 min incubation at 4 °C (−60 min), and when added immediately prior to warming (0 min) or after 10 min. However, when drugs were added at 60 or 120 min, only OXY still showed a significant inhibition higher than 50% which was lost for NIC and RAF (Fig. 4a).

To support these findings a western blotting for HAdV E1A protein was run at 6 h post infection. Results obtained showed a significantly lower expression of E1A protein in samples treated with NIC and RAF compared with OXY (Fig. 4b).

### Impact on HAdV genome accessibility to the nucleus

**Figure 5:**
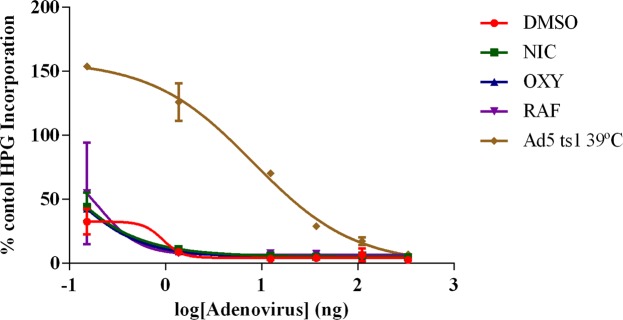
Impact on HAdV endosomolysis. NIC, OXY and RAF did not attenuate endosomolysis. Data shows the percentages of L-homopropargylglycine (HPG) incorporation. Results represent means ± SD of triplicate assays.

This assay evaluated the impact of the salicylanilide anthelmintic drugs on HAdV endosomal escape. After the binding and internalization of the HAdV viral particles, the exposure of protein VI inside the endosome triggers endosomolysis and the partially decapsidated HAdV escape to the cytosol from where it is transported to the nuclear pore complex at the nuclear membrane by the microtubule network. We used a functional assay comprising HAdV-mediated co-delivery of α-sarcin in cells as a marker of the ability of these salicylanilide drugs to alter virus-mediated endosomolysis^19,20^. No significant differences in the ID_50_ (50% inhibitory doses) for HAdV-mediated endosome penetration were detected in the presence of any of the salicylanilide drugs (Fig. 5) or the DMSO negative control. In contrast, the control virus Ad5*ts*1, an endosome penetration-defective mutant, exhibited a significant increase in the ID_50_ compared to the DMSO control (Fig. 5).

**Figure 6:**
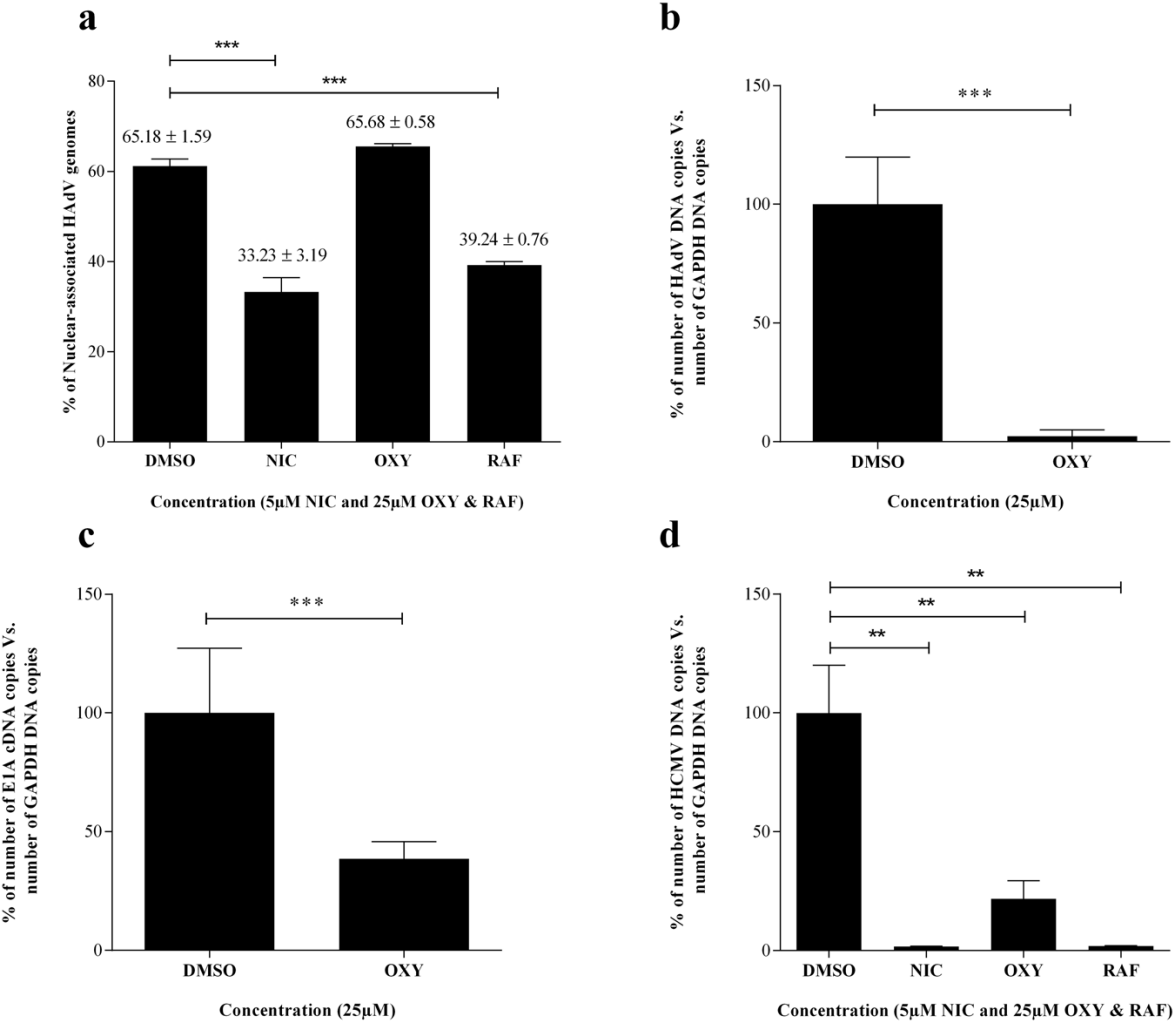
Effect of OXY on HAdV and HCMV DNA replication. The presence of NIC and RAF interfered with HAdV genomes access to the nucleus whilst OXY did not. Bars represent means ± SD of triplicate samples from two independent experiments (**a**). OXY decreased the *de novo* production of HAdV DNA copies significantly compared to a positive control 24 hours post-infection in a quantitative PCR assay (**b**), and significantly reduced expression of immediate early gene E1A compared to a negative control 6 hours post-infection in a quantitative PCR assay (**c**). Furthermore, NIC, OXY and RAF reduced *de novo* production of HCMV DNA copies 72 hours post-infection in a quantitative PCR assay (**d**). Results are expressed as the relative copy number of HAdV DNA, HAdV E1A mRNA or HCMV DNA normalized to GAPDH copy number and are presented as the mean ± SD from triplicate assays.

Once viral particles leave the endosome they are transported to the nuclear pore complex where HAdV genomes are imported into the nucleus^21^. We hypothesized that if any of the drugs examined was blocking a step in the HAdV entry, as expected for NIC, the number of genomes that reach the nucleus would be lower. We next evaluated the ability of these salicylanilide drugs to block HAdV genome accessibility to the nucleus by the quantification of HAdV genomes isolated from the nucleus^22^. As reflected in Fig. 6a, only NIC and RAF showed a significant block in the accessibility of HAdV genomes to the nucleus (Fig. 6a). The DNA copy number of the cellular gene GAPDH was also evaluated in both the nucleus and the cytoplasm as a control for the purity of nuclear isolation (data not shown). Since our results indicated that NIC and RAF hindered the accessibility of HAdV genome to the nucleus while OXY did not, we resolved to determine if this anthelmintic drug affected later steps in HAdV life cycle.

### Impact on HAdV replication

The next step was to examine the effect that OXY had upon efficient HAdV DNA replication using quantitative real-time PCR (qPCR). A549 cells were infected with HAdV and incubated for 2 h at 37 °C. Then, the unbound virions were washed-out and cell cultures were incubated for 24 h. HAdV DNA was extracted at that point to limit the interference caused by subsequent rounds of infection occurring 32–36 h post-infection^23^. We used quantitative PCR to quantify in a single round of infection the synthesis of new HAdV DNA copies as a measure of DNA replication efficiency. The presence of OXY (25 μM) significantly inhibited HAdV5 DNA replication by more than 50% (*p* < 0.05, Dunnett’s Multiple Comparison test), whilst the quantification of the DNA copies of the GAPDH gene did not showed a significant effect (Fig. 6b). This reduction in the HAdV DNA copy number at the nucleus in the presence of OXY implicated two alternatives for its mechanism of action: i) OXY could inhibit HAdV DNA replication by interfering with a protein required in this process like HAdV DNA polymerase, DNA binding protein (DBP) or terminal protein (TP) or, alternatively, ii) OXY could impact transcription of the HAdV immediate early gene E1A, which is a key step before DNA replication. To assay the inhibition of HAdV mRNA transcription, we infected A549 cells in the presence of OXY (25 μM) for 6 h. After the infection, we quantified the RNA copy number of the E1A gene using quantitative reverse transcription (RT-PCR). As shown in Fig. 6c, OXY exerted a significant decrease in the E1A mRNAs copy number compared with the control treated with DMSO.

### Salicylanilide anthelmintic drugs restrict HCMV infection

Based on previous works where piperazine derivatives and nucleotide and nucleoside analogues showed antiviral activity against multiple dsDNA virus including cytomegalovirus (HCMV) and HAdV^24,25^, we evaluated the inhibitory activity of NIC, OXY and RAF on HCMV DNA replication. The presence of these anthelmintic drugs generated significant reductions in the quantification of total HCMV DNA 72 h after infection of MRC-5 cells (Fig. 6d). OXY showed a 79% decrease whilst NIC and RAF reached a 98.3% and a 98.1% decrease, respectively of HCMV DNA replication, showing no significant differences between samples for the quantification of the GAPDH gene (data not shown).

### Salicylanilide anthelmintic drugs combination improves their antiviral activity

Since we found different mechanisms of action for these three drugs we hypothesize that their combination should significantly improve their antiviral activity. To evaluate our hypothesis we conducted a combination study based on the Chou-Talalay method for drug combination using the CalcuSyn software^26,27^. The constant ration for each combination was selected based on the IC_50_ values for each drug. The data for all the combinations showed good conformity to the mass action law (r = 0.978–0.999) (Table 2). All the combinations were classified as synergistic at the different ratios used and at all inhibitory levels analyzed. The NIC:OXY (ratio 1:4) and the NIC:OXY:RAF interactions at IC_50_, IC_75_ and IC_90_ levels of inhibition were classified as strong synergism (Table 2). For the NIC:RAF (ratio 1:2) combination the IC_50_ and IC_75_ levels of inhibition were classified as synergism and the IC_90_ level as strong synergism (Table 2). Finally, the combination RAF:OXY (ratio 1:2) was classified as synergism at all the levels of inhibition (Table 2).

**Table 2:** CalcuSyn output for the different combinations of the three anthelmintic drugs.

Drug (ratio)	Combinatory index (CI) values at			r
	IC_50_	IC_75_	IC_90_	
NIC + OXY (1:4)	0.229	0.215	0.204	0.997
NIC + RAF (1:2)	0.373	0.322	0.280	0.992
RAF + OXY (1:2)	0.748	0.597	0.490	0.978
NIC + OXY + RAF (1:4:2)	0.244	0.158	0.100	0.999

The combinatory index values are shown for the combinations at the IC_50_, IC_75_ and IC_90_ levels of inhibition. The r value for each combination is also reported to indicate the correlation coefficient of the data to the mass action law.

## Discussion

The aim of this study was to evaluate the anti-HAdV activity of NIC, a salicylanilide anthelmintic drug of human use to set the basis for its further experimental and clinical development as a potential new treatment for HAdV infections. Moreover, we included in our evaluation other two chlorinated salicylanilide anthelmintic derivatives approved for animal use, OXY and RAF, because of their closely related structure and mechanism of action with NIC. It is well-known that these drugs act as uncouplers of the oxidative phosphorylation, involving dissipation of the membrane potential^28^. In case of NIC, Jurgeitr *et al*.^14^ previously demonstrated that its antiviral mechanism of action was specifically associated with the neutralization of the endosomal pH, thus showing a broad antiviral activity against pH-dependent viruses. Although HAdV endosomal escape is not exclusively dependent of pH, a low pH has proven to be associated with the endosomal escape of HAdV^20,29^. With these premises, we initially assumed a probable common anti-HAdV mechanism of action for these three drugs and decided to characterize their anti-HAdV activity as a previous step before the evaluation of their safety and efficacy in the Syrian hamster model of HAdV infection.

Here, we show that NIC, OXY and RAF exert significant anti-HAdV and anti-HCMV activity at low concentrations. To put this data into perspective, it is noteworthy that the reported IC_50_ values for cidofovir, the drug of choice for the treatment of HAdV infections, are higher than those shown by these three salicylanilide drugs^30,31^. In addition, following our own methodologies, the IC_50_ value for cidofovir was 24.06 ± 5.9 µM, significantly higher than the values obtained for the three salicylanilide drugs. As for their mechanism of action, the data obtained from this work demonstrates that one of these three closely-related drugs did not share the expected mode of action. We have confirmed in our work that NIC, and as a novelty RAF, acts to decrease the number of HAdV genomes associated with the host nucleus, however, as demonstrated by our physiologic assay using the ribotoxin α-sarcin NIC and RAF may act in a later step after HAdV endosomal escape. Unlike NIC and RAF, OXY did not inhibit the access of HAdV genomes to the nucleus. This observation was confirmed in their time-course assay where we found that, for NIC and RAF inhibition occurred at earliest times of the HAdV replicative cycle than for OXY as well as in the western-blot assay at 6 hpi that showed higher inhibition of the E1A gene expression for NIC and RAF. As shown by our results, the high inhibition in the HAdV DNA replication generated by OXY may be the consequence of the previous and significant inhibition of the E1A gene transcription, which is supported by our results showing a clear inhibition of the E1A transcription at 6 hpi, both by quantitative real-time PCR and by western-blot. It is well known that prior to HAdV DNA replication, transcription of E1A by cellular RNA polymerase II takes place from the E1A promoter^32^. Then, E1A protein promotes its own transcription and is needed for the subsequent expression of the early genes E1B, E2, E3 and E4 from different promoters as well as for DNA replication and that is probably why we observed that significant inhibition on HAdV DNA replication. The existence of different mechanisms for the antiviral activity of these three drugs is supported by the significant combinatory index values obtained using the CalcuSyn software for all the drug combinations (Table 2). The three anthelmintic drugs showed a moderate synergistic to a strong synergistic activity when they were combined in pairs, which was especially strong at all the levels of inhibition evaluated for the NIC-OXY combination. Likewise, using the combination of the three of them they showed a strong synergistic activity at all the levels of inhibition evaluated.

Repositioning of drugs for diseases different than those they were approved for is a valuable alternative for drug discovery and development since it reduces significantly the high cost and the time-consumption of developing a new drug^33–36^. In case of NIC and other anthelmintic drugs, numerous reports support their repurposing as anti-cancer drugs due to their cancer inhibitory properties^37–40^. NIC is an FDA-approved drug that has been used for many years to treat helminthic infections in humans and has previously demonstrated its antiviral potential against different viruses such as Japanese encephalitis flavivirus, zika virus, coronavirus, human rhinovirus or influenza virus^14–17^. In rats, NIC is known to exert low *in vivo* toxicity, with a median lethal dose 50% (LD_50_) of 5,000 mg/kg body weight and generate peak serum concentrations between 1.08 µM and 25 µM, depending on the dose and the administration route^40,41^. In healthy humans orally treated with 750–2,000 mg NIC per person, no signs of intoxication were seen^42^, and the drug remained detectable for 1-2 days. Peak serum concentrations between 0.8–18 µM were reported^42,43^. As shown in our results, these serum concentrations will be enough to reach the IC_50_ for HAdV (Table 1).

OXY and RAF are other FDA-approved drugs for veterinary use to treat helminthic infections. For OXY, high serum concentrations ranging from 12.26 µM to 41.69 µM have been reported in sheep and goats after a single oral dose of 15 mg/kg^44^. In the case of RAF, an intravenous single dose of 10 mg/kg in goats generated serum concentrations ranging from 2.8 µM to 139.98 µM registered at 0.08 h and 12 h, respectively^45^. After a single oral dose of 22.5 mg/kg also in goats, the maximum serum concentrations obtained were 49.33 µM and 21.40 µM at 36 h and 168 h, respectively^45^. In both cases, OXY and RAF serum concentrations are significantly higher than their IC_50_ for HAdV (Table 1). As for their toxicity, LD_50_ of RAF has been reported to be higher than 2,000 mg/kg of body weight in rats while for OXY, according to the European Medicines Agency (EMEA), this LD_50_ would be higher than 3,000 mg/kg of body weight (also in rats)^46^.

Our findings show a significant anti-HAdV activity for the three salicylanilide drugs targeting different steps on the HAdV life cycle. NIC and RAF would mainly block HAdV infection at some point between endosomal escape and DNA release into the nucleus, whilst the activity of OXY would be specifically targeting HAdV immediately early gene E1A transcription. Our findings support the evaluation of these three drugs in the Syrian hamster model of HAdV infection to evaluate their efficacy and safety. The pharmacokinetic profile of these salicylanilide drugs showing low water solubility and oral bioavailability may hamper its further clinical development^39^. In this situation, the generation of derivatives of these salicylanilide drugs based on rational chemical approaches may improve their poor pharmacokinetics, increasing their solubility and bioavailability as potential clinical candidates for antiviral therapy.

## Methods

### Cells and virus

Human A549, 293 and MRC-5 cell lines were obtained from the American Type Culture Collection (ATCC, Manassas, VA). The 293β5 stable cell line was generated by transfecting the human β5 gene into 293 cells^47^. These cell lines were propagated in Dulbecco’s modified Eagle medium (DMEM, Life Technologies/Thermo Fisher) supplemented with 10% fetal bovine serum (FBS) (Omega Scientific, Tarzana, CA), 10 mM HEPES, 4 mM L-glutamine, 100 units/ml penicillin, 100 μg/ml streptomycin, and 0.1 mM non-essential amino acids (complete DMEM).

Wild-type HAdV5 (species C), HAdV16 (species B), HAdV19 (species D), and human cytomegalovirus (HCMV) AD169 were obtained from the ATCC. The HAdV5-GFP and HAdV16-GFP used in this study are replication-defective viruses with a CMV promoter-driven enhanced green fluorescent protein (eGFP) reporter gene cassette in place of the E1/E3 region^48^. HAdV were propagated in 293β5 cells and isolated from the cellular lysate by cesium chloride (CsCl) density gradient combined with ultracentrifugation. Virus concentration in mg/mL was calculated using the Bio-Rad Protein Assay (Bio-Rad Laboratories) and converted to virus particles/mL (vp/mL) using 4 × 10^12^ vp/mg.

### Cytotoxicity assay

NIC, OXY and RAF cytotoxicity was evaluated using the Alamar Blue Cell Viability Assay (Invitrogen) according to the manufacturer’s instructions. Actively dividing A549 or 293β5 cells were incubated with the salicylanilide anthelmintic drugs for 48 h. After this incubation, the Alamar Blue Reagent was added to the cells (1/10th Alamar Blue Reagent in culture medium) for an extra 4 h. Their 50% cytotoxic concentration (CC_50_) was calculated as previously reported by Cheng *et al*.^49^. The selectivity index (SI) was calculated as the ratio of CC_50_ to IC_50_, where the IC_50_ is defined as the concentration of the anthelmintic drug that inhibits HAdV infection by 50%.

### HAdV plaque assay

Anthelmintic drugs were tested in a dose-response assay using an MOI of 0.06 vp/cell and drug concentrations ranging from 10 to 0.1 μM in a plaque assay. Briefly, a density of 4 × 10^5^ 293β5 cells per well were seeded in 6-well plates. At 80–90% confluency they were infected with HAdV5-GFP or HAdV16-GFP (0.06 vp/cell) and rocked for 2 h at 37 °C. After this incubation wells were washed-out with PBS. Then, cells were carefully overlaid with 4 mL/well of equal parts of 1.6% (water/vol) Difco Agar Noble (Becton, Dickinson & Co., Sparks, MD) and 2× EMEM (Minimum Essential Medium Eagle, BioWhittaker) supplemented with 2 × penicillin/streptomycin, 2× L-glutamine, and 10% FBS. The mixture also contained the drugs in concentrations ranging from 10 to 0.15 μM. After incubation for 7 days at 37 °C, virus plaques were scanned with a Typhoon 9410 imager (GE Healthcare Life Sciences), and quantified with ImageJ^50^.

To determine the time course of the three anthelmintic drugs-mediated inhibition, parallel samples of wild-type HAdV5 were incubated with or without 5 μM of NIC and 25 μM of RAF or OXY in complete DMEM at 4 °C for 1 h. Virus (100 vp/cell) was then added to A549 cells (150,000 cells/well in a 24-well plate) and incubated at 37 °C. Anthelmintic drugs were added at the indicated time points (−60, 0, 10, 60, 120 and 360 min) before or during this incubation. After incubation at 37 °C and 5% CO_2_ for 24 h DNA was purified from the cell lysate using the QIAamp DNA Mini Kit (QIAGEN, Valencia, CA) following the manufacturer’s instructions, and the DNA was quantified by quantitative PCR following the above described protocol.

### Analysis of anthelmintic drug combinations

The software packet Calcusyn (BioSoft, Ferguson, MO, USA), which compares the drug concentrations required in combination to generate a given effect to the drug concentration that would be needed individually to achieve that same effect was used. For this assay a plaque dose-response assay was carried out using all the possible combination of the three drugs starting from twice the IC_50_ obtained previously for each compound and the ratio of those concentrations. CalcuSyn software interpolates the drugs concentrations needed in combination at the selected ratio to generate effects of 50%, 75% and 90% inhibition and compares these combined drug concentrations with the concentrations from the three drugs’ individual dose-response curves required to achieve the same inhibition. The combination effect of the three drugs was reported by the combination index (CI) value, a quantitative estimation of the pharmacological interaction which uses the potency (IC_50_) and the shape of the dose-response curve of each individual drug and their combinations. The CI value was interpreted in accordance with Matthews *et al*.^27^.

### Nuclear-associated HAdV genomes

The nuclear accessibility of HAdV genomes was evaluated by real-time PCR following a previously described protocol with a few modifications^22^. Briefly, 1 × 10^6^ A549 cells in 6-well plates were infected with wild-type HAdV5 (MOI 2,000 vp/cell) in the presence of 5 μM NIC or 25 μM OXY and RAF, or the same volume of DMSO for negative control. Forty-five minutes after infection, A549 cells were trypsinized and collected, and then washed twice with PBS. Then, nuclear and cytoplasmic fractions were separated using a hypotonic buffer solution consisting of 20 mM Tris-HCl pH 7.4, 10 mM NaCl, and 3 mM MgCl_2_. The cell pellet was resuspended in 500 μL of 1 × hypotonic buffer and incubated for 15 min at 4 °C. Then, 25 μL of NP-40 was added and the samples were vortexed. The homogenates were then centrifuged for 10 min at 835 g at 4 °C. HAdV DNA was isolated from the nuclear (pellet) and the cytoplasmic fractions (supernatant) using the QIAamp DNA Mini Kit (QIAGEN, Valencia, CA).

### HAdV-mediated endosome disruption

To measure endosome disruption, HAdV-mediated ribotoxin (α-sarcin) delivery assays were performed as previously described^51^. Three-fold serial dilutions (333–0.15 ng) of Ad5ts1 or wild-type HAdV5 were preincubated with cells in the presence of 5 μM NIC or 25 μM OXY and RAF, or the same volume of DMSO (negative control) for 1 h on ice. Then, the medium was removed and replaced with 50 ml DMEM-containing 0.1 mg/ml of α-sarcin (Calbiochem/EMD Biosciences, La Jolla, CA) and the virus/drug mixtures, and incubated for 2 h at 37 °C. The Click-iT HPG Alexa Fluor 488 Protein Synthesis Assay Kits (Invitrogen) was used to analyse protein synthesis according to the manufacturer’s instructions. The incorporation of the amino acid analog of methionine L-homopropargylglycine (HPG) containing Alexa Fluor 488 azide was measured using a Typhoon 9410 imager (GE Healthcare Life Sciences) and calculated subtracting the background level of the control well containing L-homopropargylglycine (HPG) and α-sarcin but not virus (100% incorporation).

### HAdV DNA and mRNA quantification by real-time PCR

For DNA quantification, 150,000 A549 cells/well in a 24-well plate were infected with 100 vp/cell (wild-type HAdV5) and incubated for 2 h at 37 °C in complete DMEM. Then, the excess of virus was washed-out, and the medium was replaced with 500 μL of complete DMEM containing 50 μM of either anthelmintic drugs or the same volume of DMSO (negative control). All samples were done in triplicate. After 24 h of incubation at 37 °C and 5% CO_2_, DNA was purified from the cell lysate using the QIAamp DNA Mini Kit (QIAGEN, Valencia, CA) following the manufacturer’s instructions. TaqMan primers, probes and PCR conditions were like those previously reported^51^.

For the evaluation of RNA expression, same conditions of infection applied for the DNA quantification were used. Six hours after infection, RNA was purified with the miRCURY RNA Isolation Kit (Exiqon Inc., MA) following the manufacturer’s instructions. Quantification of RNA copy numbers was performed using primers in conditions previously reported for E1A^52^.

Human glyceraldehyde-3-phosphate dehydrogenase (GAPDH) gene was used as internal control. Primers, probes and conditions applied for GAPDH were those previously reported by Rivera *et al*.^52^. For quantification, gene fragments of hexon and GAPDH were cloned into the pGEM-T Easy vector (Promega), and known concentrations of those vectors were used to generate a standard curve for each experiment. All assays were performed in a C1000 thermal cycler apparatus (BioRad).

### Virus yield reduction

A burst assay was used to assay the effect of the three anthelmintic drugs on virus production. A549 cells were infected with wild-type HAdV5, wild-type HAdV16 or wild-type HAdV19 in the presence or absence of 5 μM NIC or 25 μM OXY and RAF. After incubation for 48 h, cells were harvested and subjected to three rounds of freeze/thaw. Then, serial dilutions of clarified lysates were titrated on A549 cells, and TCID_50_ values were calculated using a previously reported end-point dilution method^53^.

### HCMV infectivity assay by quantitative PCR

To test the anti-HCMV activity of these anthelmintic drugs, MRC-5 cells were seeded in a 6-well plate (1.75 × 10^5^ cells/well), infected with HCMV (MOI of 0.05 vp/cell) and incubated in complete DMEM in the presence of 5 μM NIC or 25 μM in case of OXY and RAF or the same volume of DMSO in triplicate. Then, cells were incubated for 72 h at 37 °C and 5% CO_2_ and HCMV DNA was purified from the cell lysate using the QIAamp DNA Mini Kit (Qiagen, Valencia, CA) following the manufacturer’s instructions. Real-time PCR primers, mixtures and protocols were the same as previously reported^25^.

### Statistical Analyses

Statistical analyses were carried out using the GraphPad Prism 6 suite. Data are presented as the mean of triplicate samples ± standard deviation (SD), unless otherwise indicated. *P* < 0.05 was considered statistically significant.

## Supplementary information

Supplementary Figure S1
