# Supplementary material for: Repositioning salicylanilide anthelmintic drugs to treat adenovirus infections
Source: Sci Rep. 2019 Jan 9;9:17. doi: 10.1038/s41598-018-37290-3 (PMC6327057; doi:10.1038/s41598-018-37290-3)
Supplement: Supplementary file 1 — Supplementary Figure S1 [file 41598_2018_37290_MOESM1_ESM.pdf]

SUPPLEMENTARY INFORMATION

Repositioning salicylanilide anthelmintic drugs to treat adenovirus infections

José A. Marrugal-Lorenzo, Ana Serna-Gallego, Judith Berastegui Cabrera, Jerónimo Pachón, and Javier Sánchez-Céspedes

Supplementary Figures and Legends

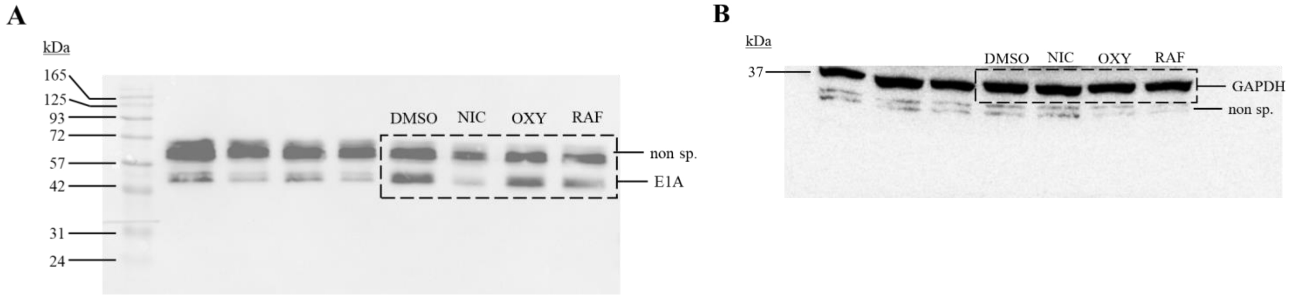

Supplementary Figure S1: Full-length Western blotting film scans. Full-length scans for (A) and (B) Western blotting films used to generate **Fig. 4b**. In **Fig.** Dashed-line boxes indicate cropped areas used for the main figure. Molecular weights (MW) in kDa are indicated on the side of the scans. Specific signals corresponding to the proteins of interest are indicated by a line. Non-specific signals due to antibody cross-reactivity are indicated by a line with the label "*non-sp.*".
